# Protocol for preparing SUV420H1 in complex with the nucleosome containing H2A.Z and H4K20Ecx for structure determination

**DOI:** 10.1016/j.xpro.2024.103295

**Published:** 2024-09-06

**Authors:** Li Huang, Zheng Zhou

**Affiliations:** 1 National Laboratory of Biomacromolecules, Institute of Biophysics, Chinese Academy of Sciences, Beijing 100101, China; 2 Key Laboratory of Epigenetic Regulation and Intervention, Institute of Biophysics, Chinese Academy of Sciences, Beijing 100101, China; 3 University of Chinese Academy of Sciences, Beijing 100049, China

**Keywords:** Cryo-EM, Protein Biochemistry, Protein expression and purification

## Abstract

The histone lysine methyltransferase SUV420H1 preferentially targets the H2A.Z-containing nucleosome core particle (H2A.Z-NCP) and catalyzes the H4K20me2 modification at replication origins. Here, we present a protocol for preparing SUV420H1 in complex with the nucleosome containing H2A.Z and H4K20Ecx for structure determination. We describe steps for the installation of S-ethyl-cysteine (Ecx), nucleosome and complex preparation, and performing the cryoelectron microscopy (cryo-EM) sample check. This protocol substitutes lysine 20 in histone H4 with S-ethyl-cysteine (H4K20Ecx), which enhances the stability of the interaction between SUV420H1 and nucleosomes.

For complete details on the use and execution of this protocol, please refer to Huang et al.^1^

## Highlights

• Universal approach to integrate Ecx in histones with low yield
• Detailed steps for installing S-ethyl-cysteine (Ecx) into histone H4K20C
• Extensive protocol detail for cryo-EM nucleosome complex preparation

## Abstract (editor highlights)

Publisher’s note: Undertaking any experimental protocol requires adherence to local institutional guidelines for laboratory safety and ethics.

## Graphical abstract

**Figure:**
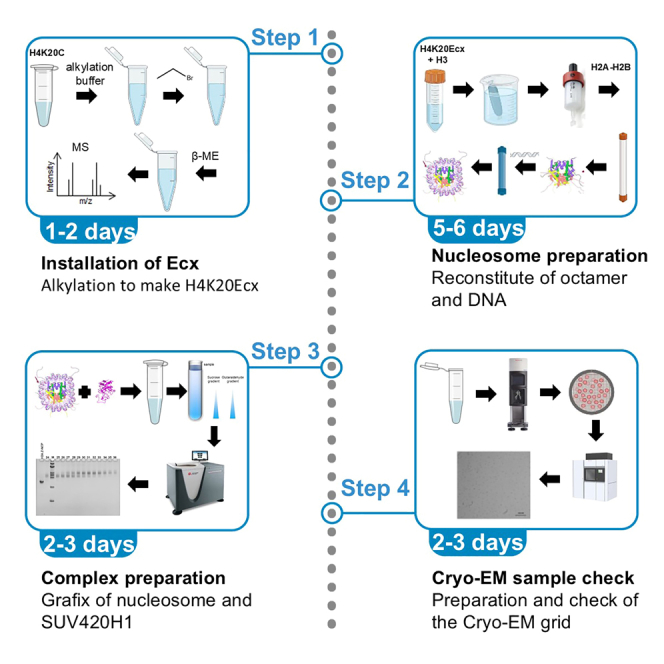

## Before you begin

Studies have shown that substituting histone H3 lysine with the non-native amino acid norleucine (Nle) enhances the binding stability between the nucleosome core particle and various methyltransferases.^2^^,^^3^ However, efforts to replace H4 lysine 20 with norleucine were not successful. To overcome this difficulty, we used S-ethyl-cysteine (Ecx) as a substitute at H4K20, creating H4K20Ecx. Our study indicates that H4K20Ecx might stabilize the interaction between SUV420H1 and the nucleosome, similarly to the observed effect with histone H3 with norleucine mutant.

This protocol illustrates the step-by-step preparation of SUV420H1-H2A.Z NCP^H4K20Ecx^ complexes. It includes comprehensive instructions for expressing and purifying SUV420H1, reconstituting H2A.Z nucleosomes with the H4K20Ecx mutation, and preparing SUV420H1-H2A.Z NCP^H4K20Ecx^ complexes.

### Preparation of 147-bp W601 DNA

**Timing: 5–6 days**

**CRITICAL:** After transformation, it is essential to verify the plasmid from multiple colonies using either restriction enzyme digestion. This step is crucial to ensure high yields of the desired DNA.

The large-scale production of 147-bp Widom 601 DNA, used for nucleosome reconstitution, was purified following previously described methods.^4^

1. Transform pUC19-147x12 plasmid into Top10 competent cells as per the manufacturer’s instructions.
  a. Thaw 50 μL of Top10 competent cells on ice.
  b. Add 1 μL pUC19-147x12 plasmid (about 100 ng) to competent cells, moving the pipette through the cells while dispensing. Gently tap tubes to mix.
    ***Note:*** Limit the volume of DNA to no more than one-tenth of the volume of competent cells.
  c. Incubate cells on ice for 30 min.
  d. Perform heat shock at 42°C for 90 s in a water bath.
  e. Quickly transfer the centrifuge tube to an ice bath to cool for 3 min.
  f. Add 500 μL of LB media and incubate at 37°C for 1 h in the incubator at 220 rpm.
  g. Plate 200 μL of the transformed cells evenly onto an LB agar plate with ampicillin (100 μg/mL) and incubate at 37°C for 12–18 h.
2. Pick 3–4 colonies into separate 3 mL LB medium (100 μg/mL ampicillin) and incubate at 37°C with shaking at 220 rpm for 12–18 h.
3. Extract plasmids from 2 mL cell cultures using TIANprep rapid Mini plasmid Kit.
4. Digest 200 ng of the plasmids with EcoRV enzymes according to the following system at 37°C for 3 h.

**Table:** 

DNA	200 ng
10 × H Buffer	2 μL
EcoRV	1 μL
ddH_2_O	up to 20 μL

**Figure 1:**
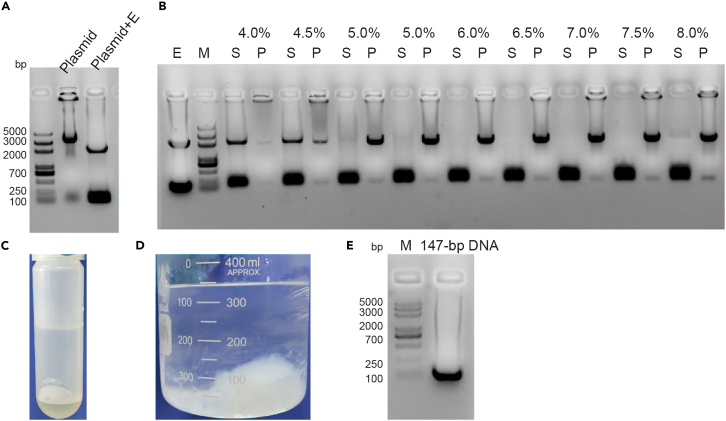
Preparation of 147-bp DNA (A) pUC19-147x12 plasmid, “plasmid+E″ means the plasmid digested by restriction enzymes EcoRV. (B) The 147-bp DNA was then isolated by PEG precipitation. “E” means the plasmid digested by restriction enzymes EcoRV. “S” means the supernatants and “P” means the pellets after PEG precipitation. (C) The picture of DNA extraction by PCIA. (D) The picture of DNA precipitate by ethanol. (E) The analysis of 147-bp DNA.

***Note:*** If multiple bands are produced, it indicates incomplete digestion.

5. Run 1% agarose gel to analyze the digestion samples. The correct plasmid should produce 147-bp DNA and pUC19 vector bands (refer to Figure 1A).

6. Select clones with verified plasmids and scale up by adding 200 μL of cell culture from step 2 into 200 mL LB medium (100 μg/mL ampicillin). Incubate at 37°C with shaking at 220 rpm for 12–18 h.
7. Add 15 mL cell cultures from step 6 into flasks with 800 mL of LB medium (100 μg/mL ampicillin), total 12 flasks. Incubate at 37°C at 220 rpm for 20 h.
8. Centrifuge the cells at 5000 g and 4°C for 30 min to harvest, then proceed with large-scale plasmid extraction as described in the published protocol.^4^ The plasmid from 12 L of cell culture is typical 100–150 mg.

**Pause point:** The purified pUC19-147x12 plasmid can be stored at −80°C for up to 2 years.

9. Digest the plasmids in large quantities using EcoRV enzymes as per the following protocol. Taking the digestion of 20 mg of DNA as an example.

**Table:** 

DNA (2 mg/mL)	10 mL
10 × H Buffer	2 mL
EcoRV (100 U/μL)	0.12 mL
dd H_2_O	up to 20 mL
Total	20 mL

  a. Add DNA according to the concentration to achieve a final concentration of 1 μg/μL and add 1/10 volume of 10×H buffer.
  b. Add Takara EcoRV enzyme to achieve a final concentration of 0.6 U/μg, and finally add TE10/0.1 (10 mM Tris–HCl pH 8.0, 0.1 mM EDTA) buffer to make up the remaining volume according to the following system.
  c. Incubate at 37°C for 16 h.
10. Examine the EcoRV-digested plasmid on a 1% agarose gel. If multiple bands appear, indicating incomplete digestion, add 50% more enzyme and incubate for another 18 h.
11. Upon complete digestion, test the experimental conditions of removing the plasmid backbone with a DNA concentration of 1 mg/mL.
  a. Precipitate the plasmid backbone using 40% PEG6000 to a final concentration of 4–8% PEG 6000 and use 4 M NaCl to bring the final concentration of NaCl to 0.5 M.

**Table:** 

	PEG6000 final concentration (%) (μL)								
	4	4.5	5	5.5	6	6.5	7	7.5	8
1 mg/mL DNA	10	10	10	10	10	10	10	10	10
4 M NaCl	6.25	6.25	6.25	6.25	6.25	6.25	6.25	6.25	6.25
40% PEG	5.00	5.63	6.25	6.88	7.50	8.13	8.75	9.38	10.00
TE10/0.1	28.75	28.13	27.50	26.88	26.25	25.63	25.00	24.38	0.75
Total									50 (μL)
  b. Incubate of the mixture at 37°C for 30 min and then put the tubes on ice for 30 min. Centrifuge at 31000 g at 4°C for 30 min to pellet the plasmid backbone DNA.
  c. Resuspend the precipitate in 50 μL TE 10/0.1 (10 mM Tris–HCl pH 8.0, 0.1 mM EDTA) buffer and analyze the pellets and supernatants by running a 1% agarose gel.
  d. The plasmid backbone was precipitated at 6.5% PEG 6000 conditions as shown in Figure 1B.
12. Mix the digestion samples with 6.5% PEG 6000 and 0.5 NaCl as before. Incubate of the mixture at 37°C for 30 min, and then put the tubes on ice for 30 min. Centrifuge at 31000 g at 4°C for 30 min to pellet the plasmid backbone DNA and collect the supernatant.
13. Add 1/5 volume of PCIA (phenol: chloroform: isoamyl alcohol, 25:24:1) to the DNA, and shake gently to mix, then centrifuge at 4°C, 31000 g for 15 min. Carefully remove the upper aqueous phase to new tubes and discard the discard the lower organic phase with enzyme and PEG 6000 (Figure 1C).
14. Precipitate the DNA using ethanol: Add 2.5 times the DNA volume of ice-cold anhydrous ethanol and 1/10 volume of ice-cold 3 M NaAc (pH 5.2). Place the mixture at −20°C for at least 2 h, during which some white precipitate forms (Figure 1D). Then centrifuge at 4°C, 31000 g for 30 min and discard the supernatant.
15. Dissolve the DNA pellets in an appropriate volume of TE 10/0.1 (10 mM Tris–HCl pH 8.0, 0.1 mM EDTA) buffer. Ensure the final 147-bp DNA concentration is at least 2 mg/mL. Typically, yields are 40% of the total plasmid.
16. Analyze the 147-bp DNA by running a 1% agarose gel (Figure 1E) and store the 147-bp DNA at −80°C for further use.

**Pause point:** The purified 147-bp DNA can be stored at −80°C for up to 2 years.

### Purification of histone H2A.Z/H2B

**Timing: 5–6 days**

17. Histone H2A.Z and H2B were subcloned into the expression plasmids pQlink featuring a 6×His tag at the N-terminal of H2A.Z.^5^
  a. The H2A.Z coding sequence was cloned into pQLinkH and the H2B coding sequence into pQLinkN using BamHI and HindIII restriction sites.
  b. Digest 200–500 ng of pQLinkH-H2A.Z plasmid with SwaI in a 10 μL reaction at 25°C. Similarly, digest pQLinkN-H2B with PacI in a 10 μL reaction at 37°C. After digestion, inactivate the enzymes by incubating at 65°C for 20 min, then place the tubes on ice to cool.
  c. Treat 10 μL of the DNA with 1 μL T4 DNA polymerase in a 20 μL reaction mixture (containing 1×buffer 2, 5 mg/mL BSA, and 2.5 mM dCTP for the PacI digest or 2.5 mM dGTP for the SwaI digest). Incubate for 60 min at 25°C, then heat inactivate at 65°C for 20 min.
  d. Mix the two plasmids, heat to 65°C, and then cool to 25°C for 30 min to allow for annealing.
  e. Add 2 μL of 25 mM EDTA to stop the reaction and transform the mixture into Top10 competent cells.
  f. Screen the transformants using primers pQTEV3U (5′-TATAAAAATAGGCGTATCACGAGG-3′) and pQTEV3L (5′-CCAGTGATTTTTTTCTCCATTTT-3′), which are positioned upstream of the LINK1 and downstream of the LINK2 sequences, respectively. The expected inserts are both H2A.Z and H2B. Select clones for sequencing.
18. Introduce 1 μL of pQlinkH-H2A.Z-N-H2B plasmid into 50 μL of BL21(DE3) RIPL cells, as the guidelines in step 1.
19. Pick a single colony into 100 mL LB medium with ampicillin (100 μg/mL) and chloramphenicol (34 μg/mL). Incubate at 37°C with shaking at 220 rpm for 12–18 h.
20. Grow 4.8 L LB in six bottles. Distribute 800 mL LB containing ampicillin (100 μg/mL) and chloramphenicol (34 μg/mL) into 6 flasks and then dilute 15 mL cell culture into each of them.
21. Incubate the cell culture at 37°C with shaking at 220 rpm until it reaches an OD_600_ of 0.5, then cool the culture to 16°C.
22. Add 400 μL of 1 M IPTG to achieve a final concentration of 0.5 mM and induce H2A.Z-H2B expression for 18 h.
23. Centrifuge the cells at 5000 g for 30 min at 4°C, discard the supernatant, and store the pellet at −80°C for later use.

**Pause point:** The cell pellets can be stored at −80°C for up to 2 months.

24. Resuspend the entire cell pellets in 180 mL of histone lysis buffer (20 mM Tris–HCl pH 8.0, 2 M NaCl, 25 mM imidazole, 5 mM β-mercaptoethanol) and lyse using a high-pressure homogenizer for about 8 cycles (Methods video S1).

***Alternatives:*** If a high-pressure homogenizer is not available, cell lysis can be effectively achieved through sonication.

**Figure 2:**
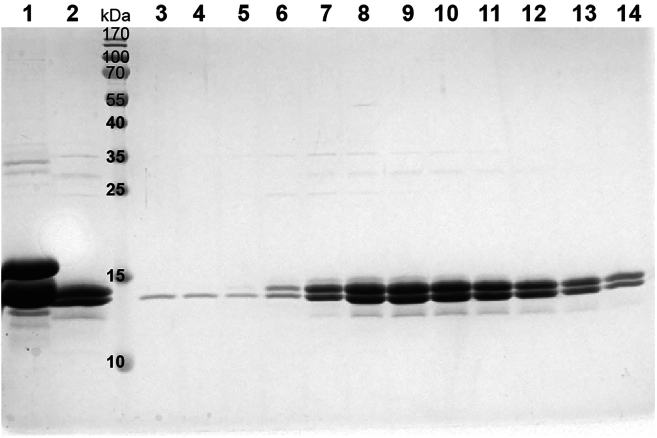
SDS PAGE analysis of H2A.Z-H2B purification Lane 1: samples with His tag. Lane 2: samples without His tag. Lane 3–14: peak fractions by SP column.
29. Add TEV Protease with 6 × his tag to the protein sample (0.06 mg TEV/ mg protein). Dialyze the protein sample against 2 L of histone lysis buffer using 3 kDa cut-off dialysis bag at 4°C for 18 h.
30. Equilibrate 1 mL of packed Ni-NTA beads with 60 mL of histone lysis buffer and reload the dialyzed sample onto equilibrated Ni-NTA beads to remove protein not cleaved and TEV Protease, wash with histone lysis buffer, and collect the flow-through samples (Figure 2, lanes 2).
31. Purify the H2A.Z-H2B protein by SP HP cation exchange column.
  a. Dilute the sample with histone buffer 0 (50 mM Tris–HCl pH 8.0, 1 mM EDTA, 5 mM β-mercaptoethanol) to lower the NaCl concentration to 500 mM NaCl.
  b. Equilibration 5 mL SP HP cation exchange column with histone buffer A with 500 mM NaCl and then load the sample into a 5 mL SP HP cation exchange column (GE Healthcare) and elute with a 0.5–2 M NaCl gradient.
  c. Check the purified protein by SDS-PAGE and collect the protein of high quality (Figure 2, lanes 7–14).
  d. Measure the protein concentration by NanoDrop 2000c and store the protein at −80°C for future use. Typically, yield of H2A.Z-H2B protein form 6 bottles is about 15–20 mg.

***Note:*** The molar ratio of histone H2A.Z to H2B in the H2A.Z-H2B dimer deviates from 1:1. Make sure the culture is cooled to 16°C and the OD_600_ of the cell culture is not higher than 0.6 at inducing.

25. Centrifuge the lysed cells at 31000 g for 1 h at 4°C. Transfer the clear supernatant with H2A.Z-H2B to a new beaker.
26. Equilibrate 3 mL of packed Ni-NTA beads with 60 mL of histone lysis buffer and mix with the clarified lysate. If using new beads, wash them with distilled water to remove the ethanol.
27. Incubate the beads and the lysate at 4°C for 1 h, then process through a gravity flow column and wash with 150 mL of histone lysis buffer.
28. Elute H2A.Z-H2B protein with about 30 mL histone elution buffer (50 mM Tris–HCl pH 8.0, 2 M NaCl, 250 mM imidazole, 5 mM β-mercaptoethanol). Run 15% SDS-PAGE to analyze the sample (Figure 2, lanes 1).

**Pause point:** the purified H2A.Z-H2B protein can be stored at −80°C for up to 1 year.

***Alternatives:*** H2A.Z protein and H2B protein can also be prepared as inclusion bodies following the detailed protocol for the purification of H4K20C/H3 proteins, and subsequently form H2A.Z-H2B dimers as described in the protocol for reconstituting (H3-H4K20C)_2_ tetramers.

Methods video S1. A video demonstrating the use of homogenizer, related to step 24 at “before you begin”

### Purification of H4K20C / H3 proteins

**Timing: 5–6 days**

The preparation of histone H4K20C / H3 proteins and nucleosomes were previously described.^4^^,^^6^

32. Introduce 1 μL of pET3a-H4K20C plasmid into 50 μL of BL21(DE3) RIPL cells as the manufacturer’s instructions in step 1.
33. Inoculate a single colony into 100 mL LB medium containing ampicillin (100 μg/mL) and chloramphenicol (34 μg/mL) and grow at 37°C with shaking at 220 rpm for 12–16 h.
34. Grow 4.8 L 2 × YT medium in six bottles. Distribute 800 mL 2×YT medium containing ampicillin (100 μg/mL) and chloramphenicol (34 μg/mL) into 6 flasks and then dilute 15 mL cell culture into each of them.
35. Incubate the cell culture at 37°C until it reaches an OD_600_ of 0.6, then add 400 mL of 1 M IPTG to induce H4K20C expression at a final concentration of 0.5 mM at 37°C for 2 h.
36. Centrifuge the cells at 5000 g for 30 min at 4°C, discard the supernatant, and store the pellet at −80°C for later use.

**Pause point:** The cell pellets can be stored at −80°C for up to 2 months.

37. Resuspend the cell pellet collected from 4.8 L LB with 180 mL histone wash buffer (20 mM Tris–HCl pH 8.0, 150 mM NaCl, 1 mM EDTA, 5 mM β-mercaptoethanol). Lyse the cells using a high-pressure homogenizer for approximately 8 cycles.
38. Centrifuge the lysed cells at 31000 g for 1 h at 4°C and discard the supernatant.
39. Resuspend the inclusion body in 150 mL histone wash buffer containing 1% Triton X-100, disaggregate using a high-speed disperser (Methods video S2), then centrifuge and discard the supernatant.

***Alternatives:*** If a high-speed disperser is not available, inclusion body can be disaggregated effectively through sonication.

40. Wash the inclusion body twice with histone wash buffer containing 1% Triton X-100 and once with the buffer alone. Collect the inclusion body.
41. Dissolve the inclusion bodies in 50 mL of histone unfolding buffer (20 mM Tris-HCl pH 8.0, 7 M guanidine hydrochloride, 5 mM β-mercaptoethanol), disaggregate the pellet with a high-speed disperser, and allow to dissolve at 25°C for 1 h.
42. Centrifuge the protein sample at 210,100 g for 30 min at 25°C and collect the supernatant.
43. Dialyze the sample against 2 L Urea buffer 0 (20 mM Tris-HCl pH 8.0, 1 mM EDTA, 8 M urea, 5 mM β-mercaptoethanol) for at least 3 h, replacing the dialysis buffer twice.

***Note:*** Guanidine hydrochloride (GuHCl) is generally considered a stronger denaturant compared to urea. GuHCl is a salt composed of guanidine and hydrochloric acid, commonly used to denature histone pellets. Guanidine hydrochloride is a salt which can affect the binding of proteins to ion exchange columns. Urea, an organic compound, which can not affect the binding of proteins to ion exchange columns, is frequently used in the purification of histones.

44. Equilibration 5 mL SP HP cation exchange column with Urea buffer 0 without NaCl and then load the sample into the 5 mL SP HP cation exchange column (GE Healthcare) and elute with a 0–2 M NaCl gradient.
45. Pool the peak fractions and dialyze thoroughly against distilled water.
46. Lyophilize the proteins and store at −80°C.
47. Prepare the histone H3 proteins using the established histone inclusion body purification protocol similar to how H4K20C proteins were made.

**Pause point:** The lyophilized H4K20C or H3 protein can be stored at −80°C for up to 2 years.

Methods video S2. A video demonstrating the use of high-speed disperser, related to step 41 at “step-by-step method details”

## Key resources table

**Table:** 

REAGENT or RESOURCE	SOURCE	IDENTIFIER
**Bacterial and virus strains**		

*TOP10* chemically competent cell	Yeasen	11801ES80
*E. coli* BL21 Codon PLUS(DE3)-RIPL	Agilent	Cat#230280

**Chemicals, peptides, and recombinant proteins**		

Bromoethane	Sigma-Aldrich	Cat# 239607
Imidazole	Amresco	CS2813
Phenol:chloroform:isoamyl alcohol 25:24:1	JSENB	JS0700
Tris	Amresco	0497
HEPES	Amresco	CS7329
EDTA	Amresco	CS6001
Sucrose	Amresco	CS5723
Triton X-100	Amresco	0649
Guanidine hydrochloride	Biosharp	E424
Sodium chloride	Hopebio	SN042
TEMED	Sigma-Aldrich	T22500
Isopropyl-b-D-thiogalactopyranoside (IPTG)	Amresco	0487
Ampicillin	Solarbio	A8180
Chloramphenicol	Amresco	0230
EcoRV	Takara	D1042C
TEV Protease (His-tag)	Zhou Lab	N/A
Agarose	Biosharp	BS081
PEG6000	Sigma-Aldrich	81255
β-Mercaptoethanol	Genview	GM195
Ethidium bromide	Sangon Biotech	A500328

**Critical commercial assays**		

Miniprep Kit	TIANGEN	A0116A
One-Step PAGE Gel Fast Preparation Kit	Vazyme	E305-C1
Ni-NTA resin	QIAGEN	Cat#30230
Hitrap SP HP	GE Healthcare	Cat#17-1151-01
Superdex 200 10/300 GL	GE Healthcare	Cat#17-5175-01
TSKgel DEAE-5PW column	Tosoh	Cat#0007574
Amicon concentrators (30K)	Millipore	Cat#UFC90302
0.22 μm membrane filter	Millipore	Cat# GSTF01300
ANTA copper grids	Yibokeji	GD-NiTiA321

**Oligonucleotides**		

The guide sequence for K219A/R220A: 5′-GACAAAATAGAATTACTGGT-3′	This paper	N/A
The primers used for K219A/R220A homologous arm amplification		
The forward primer: 5′-TTAACTTTCTCAATGTGGCTGC	This paper	N/A
The reverse primer: 5′-AAGAAACTCACACCTGAAGC	This paper	N/A
The primers used for K219A/R220A mutation		
The forward primer: 5′-GTTGCAACAAAAGAGTGGGCTGC TAATGACAAAATAGAATTACTG	This paper	N/A
The reverse primer: 5′-CAGTAATTCTATTTTGTCATTAG CAGCCCACTCTTTTGTTGCAAC	This paper	N/A

**Oligonucleotides**		

pET-17b-SUV420H1(1–393)	This paper	N/A
pQlinkH-xlH2A.Z-N-xlH2B		
pET-3a xlH3	Luger et al.^4^	N/A
pET-3a xlH4	Luger et al.^4^	N/A
pET-3a xlH4-K20C		
pET-17b SUV420H1(1–393)	This paper	N/A
pQlinkH-xlH2A.Z-N-xlH2B	This paper	N/A

**Recombinant DNA**		

pET-17b SUV420H1(1–393)	This paper	N/A
pQlinkH-xlH2A.Z-N-xlH2B	This paper	N/A
pET-3a xlH3	Luger et al.^4^	N/A
pET-3a xlH4	Luger et al.^4^	N/A
pET-3a xlH4-K20C	This paper	N/A

**Other**		

Homogenizer	ATS	N/A
High-speed disperser	Labgic	N/A
NanoDrop 2000c	Thermo Fisher Scientific	N/A
ÄKTA Purifier	GE Healthcare	N/A
Optima XPN-80 ultracentrifuge	Beckman Coulter	N/A
Vitrobot	Thermo Fisher Scientific	N/A
Talos Arctica microscope	FEI	N/A

## Materials and equipment

**Table:** LB media

Reagent	Final concentration	Amount
Tryptone	1%	10 g
Yeast Extract	0.5%	5 g
NaCl	1%	10 g
ddH_2_O	N/A	Add to 1 L

Sterilize at 121°C for 15 min, then store at 25°C. The media can be stored at 25°C for 2 weeks. Add 1.5% agar to the medium to prepare LB solid medium. The agar plates can be stored at 4°C for 2 weeks.

**Table:** 2 × YT media

Reagent	Final concentration	Amount
Tryptone	1.6%	16 g
Yeast Extract	1%	10 g
NaCl	0.5%	5 g
ddH_2_O	N/A	Add to 1 L

Sterilize at 121°C for 15 min, and store at 25°C. The media can be stored at 25°C for 2 weeks.

**Table:** Histone lysis buffer

Reagent	Final concentration	Amount
Tris (1 M) pH 8.0	20 mM	20 mL
NaCl	2 M	116.88 g
2 M imidazole pH8.0	20 mM	10 mL
β-mercaptoethanol	5 mM	350 μL
ddH_2_O	N/A	Add to 1 L

Adjust pH of medium to 8.0 at about 25°C, and store at 4°C. The buffer can be stored at 4°C for one month.

**Table:** Histone elution buffer

Reagent	Final concentration	Amount
Tris (1 M) pH 8.0	20 mM	20 mL
NaCl	2 M	116.88 g
2 M imidazole pH 8.0	250 mM	125 mL
β-mercaptoethanol	5 mM	350 μL
ddH_2_O	N/A	Add to 1 L

Adjust pH of medium to 8.0 at about 25°C, and store at 4°C. The buffer can be stored at 4°C for one month.

**Table:** Histone AB / 34 buffer A

Reagent	Final concentration	Amount
Tris (1 M) pH 8.0	20 mM	20 mL
5 M NaCl	500 mM / 850 mM	100 / 170 mL
EDTA (0.5 M) pH 8.0	1 mM	2 mL
β-mercaptoethanol	5 mM	350 μL
ddH_2_O	N/A	Add to 1 L

Adjust pH of medium to 8.0 at about 25°C, and store at 4°C. The buffer can be stored at 4°C for one month.

**Table:** Histone buffer B

Reagent	Final concentration	Amount
Tris (1 M) pH 8.0	20 mM	20 mL
2 M NaCl	200 mM	116.88 g
EDTA (0.5 M) pH 8.0	1 mM	2 mL
β-mercaptoethanol	5 mM	350 μL
ddH_2_O	N/A	Add to 1 L

Adjust pH of medium to 8.0 at about 25°C, and store at 4°C. The buffer can be stored at 4°C for one month.

**Table:** Histone wash buffer

Reagent	Final concentration	Amount
Tris (1 M) pH 8.0	20 mM	20 mL
EDTA (0.5 M) pH 8.0	1 mM	2 mL
5 M NaCl	150 mM	30 mL
β-mercaptoethanol	5 mM	350 μL
ddH_2_O	N/A	Add to 1 L

Adjust pH of medium to 8.0 at 25°C, and store at 4°C. The buffer can be stored at 4°C for one month.

**Table:** Histone unfolding buffer

Reagent	Final concentration	Amount
Tris (1 M) pH 8.0	20 mM	20 mL
guanidine hydrochloride	7 M	668.71 g
β-mercaptoethanol	5 mM	350 μL
ddH_2_O	N/A	Add to 1 L

Adjust pH of medium to 8.0 at 25°C, and store at 4°C. The buffer can be stored at 4°C for one month.

**Table:** Histone urea buffer 0

Reagent	Final concentration	Amount
Tris (1 M) pH 8.0	20 mM	20 mL
β-mercaptoethanol	5 mM	350 μL
ddH_2_O	N/A	Add to 1 L

Adjust pH of medium to 8.0 at 25°C, and store at 4°C. The buffer can be stored at 4°C for one month.

**Table:** Histone urea buffer B

Reagent	Final concentration	Amount
Tris (1 M) pH 8.0	20 mM	20 mL
Urea	8 M	480 g
5 M NaCl	2 M	116.88 g
β-mercaptoethanol	5 mM	350 μL
ddH_2_O	N/A	Add to 1 L

Adjust pH of medium to 8.0 at 25°C, and store at 4°C. The buffer can be stored at 4°C for one month.

**Table:** SUV lysis buffer

Reagent	Final concentration	Amount
MES (0.5 M) pH 6.5	20 mM	40 mL
5 M NaCl	300 mM	60 mL
Glycerol	5%	50 mL
2 M imidazole pH 8.0	20 mM	10 mL
β-mercaptoethanol	5 mM	350 μL
ddH_2_O	N/A	Add to 1 L

Adjust pH of medium to 6.5 at about 25°C, and store at 4°C. The buffer can be stored at 4°C for one month.

**Table:** SUV elution buffer

Reagent	Final concentration	Amount
MES (0.5 M) pH 6.5	20 mM	40 mL
5 M NaCl	300 mM	60 mL
glycerol	5%	50 mL
2 M imidazole pH 8.0	250 mM	125 mL
β-mercaptoethanol	5 mM	350 μL
ddH_2_O	N/A	Add to 1 L

Adjust pH of medium to 6.5 at about 25°C, and store at 4°C. The buffer can be stored at 4°C for one month.

**Table:** SUV elution buffer

Reagent	Final concentration	Amount
MES (0.5 M) pH 6.5	20 mM	40 mL
NaCl	300 mM	60 mL
2 M imidazole pH 8.0	250 mM	125 mL
β-mercaptoethanol	5 mM	350 μL
ddH_2_O	N/A	Add to 1 L

Adjust pH of medium to 6.5 at about 25°C, and store at 4°C. The buffer can be stored at 4°C for one month.

**Table:** SUV buffer 0

Reagent	Final concentration	Amount
MES (0.5 M) pH 6.5	20 mM	40 mL
5 M NaCl	200 mM	40 mL
glycerol	5%	50 mL
β-mercaptoethanol	5 mM	350 μL
ddH_2_O	N/A	Add to 1 L

Adjust pH of medium to 6.5 at about 25°C, and store at 4°C. The buffer can be stored at 4°C for one month.

**Table:** SUV buffer A

Reagent	Final concentration	Amount
MES (0.5 M) pH 6.5	20 mM	40 mL
5 M NaCl	200 mM	200 mL
glycerol	5%	50 mL
β-mercaptoethanol	5 mM	350 μL
ddH_2_O	N/A	Add to 1 L

Adjust pH of medium to 6.5 at about 25°C, and store at 4°C. The buffer can be stored at 4°C for one month.

**Table:** SUV buffer B

Reagent	Final concentration	Amount
MES (0.5 M) pH 6.5	20 mM	40 mL
5 M NaCl	1 M	200 mL
Glycerol	5%	50 mL
β-mercaptoethanol	5 mM	350 μL
ddH_2_O	N/A	Add to 1 L

Adjust pH of medium to 6.5 at about 25°C, and store at 4°C. The buffer can be stored at 4°C for one month.

**Table:** SUV superdex buffer

Reagent	Final concentration	Amount
MES (0.5 M) pH 6.5	20 mM	40 mL
5 M NaCl	300 mM	60 mL
glycerol	5%	50 mL
β-mercaptoethanol	5 mM	350 μL
ddH_2_O	N/A	Add to 1 L

Adjust pH of medium to 6.5 at about 25°C, and store at 4°C. The buffer can be stored at 4°C for one month.

**Table:** Histone refolding buffer

Reagent	Final concentration	Amount
Tris (1 M) pH 8.0	20 mM	20 mL
EDTA (0.5 M) pH 8.0	1 mM	2 mL
NaCl	2 M	116.88 g
β-mercaptoethanol	5 mM	350 μL
ddH_2_O	N/A	Add to 1 L

Adjust pH of medium to 8.0 at about 25°C, and store at 4°C. The buffer can be stored at 4°C for one month.

**Table:** TE2K buffer

Reagent	Final concentration	Amount
Tris (1 M) pH 8.0	20 mM	20 mL
2 M KCl	2 M	149.1 g
EDTA (0.5 M) pH 8.0	1 mM	2 mL
β-mercaptoethanol	5 mM	350 μL
ddH_2_O	N/A	Add to 1 L

Adjust pH of medium to 7.5 at about 25°C, and store at 4°C. The buffer can be stored at 4°C for one month.

**Table:** TE buffer

Reagent	Final concentration	Amount
Tris (1 M) pH 8.0	20 mM	20 mL
EDTA (0.5 M) pH 8.0	1 mM	2 mL
β-mercaptoethanol	5 mM	350 μL
ddH_2_O	N/A	Add to 1 L

Adjust pH of medium to 7.5 at about 25°C, and store at 4°C. The buffer can be stored at 4°C for one month.

**Table:** Complex buffer

Reagent	Final concentration	Amount
HEPES (0.5 M) pH 7.5	20 mM	40 mL
Glycerol	4%	40 mL
0.5 M DTT	2 mM	4 mL
ddH_2_O	N/A	Add to 1 L

Adjust pH of medium to 7.5 at about 25°C, and store at 4°C. The buffer can be stored at 4°C for one week.

**Table:** Low sucrose buffer

Reagent	Final concentration	Amount
HEPES (0.5 M) pH 7.5	20 mM	2 mL
Sucrose	5%	2.5 g
0.5 M DTT	2 mM	200 μL
ddH_2_O	N/A	Add to 50 mL

Adjust pH of medium to 8.0 at about 25°C, and store at 4°C. This buffer should be freshly prepared.

**Table:** High sucrose buffer

Reagent	Final concentration	Amount
HEPES (0.5 M) pH 7.5	20 mM	2 mL
Sucrose	20%	10 g
0.5 M DTT	2 mM	200 μL
ddH_2_O	N/A	Add to 50 mL

Adjust pH of medium to 7.5 at about 25°C, and store at 4°C. This buffer should be freshly prepared.

***Note:*** The β-mercaptoethanol or DTT should be added before the use of the buffer.

## Step-by-step method details

### Preparation of SUV420H1

**Timing: 1.5 weeks**

**Figure 3:**
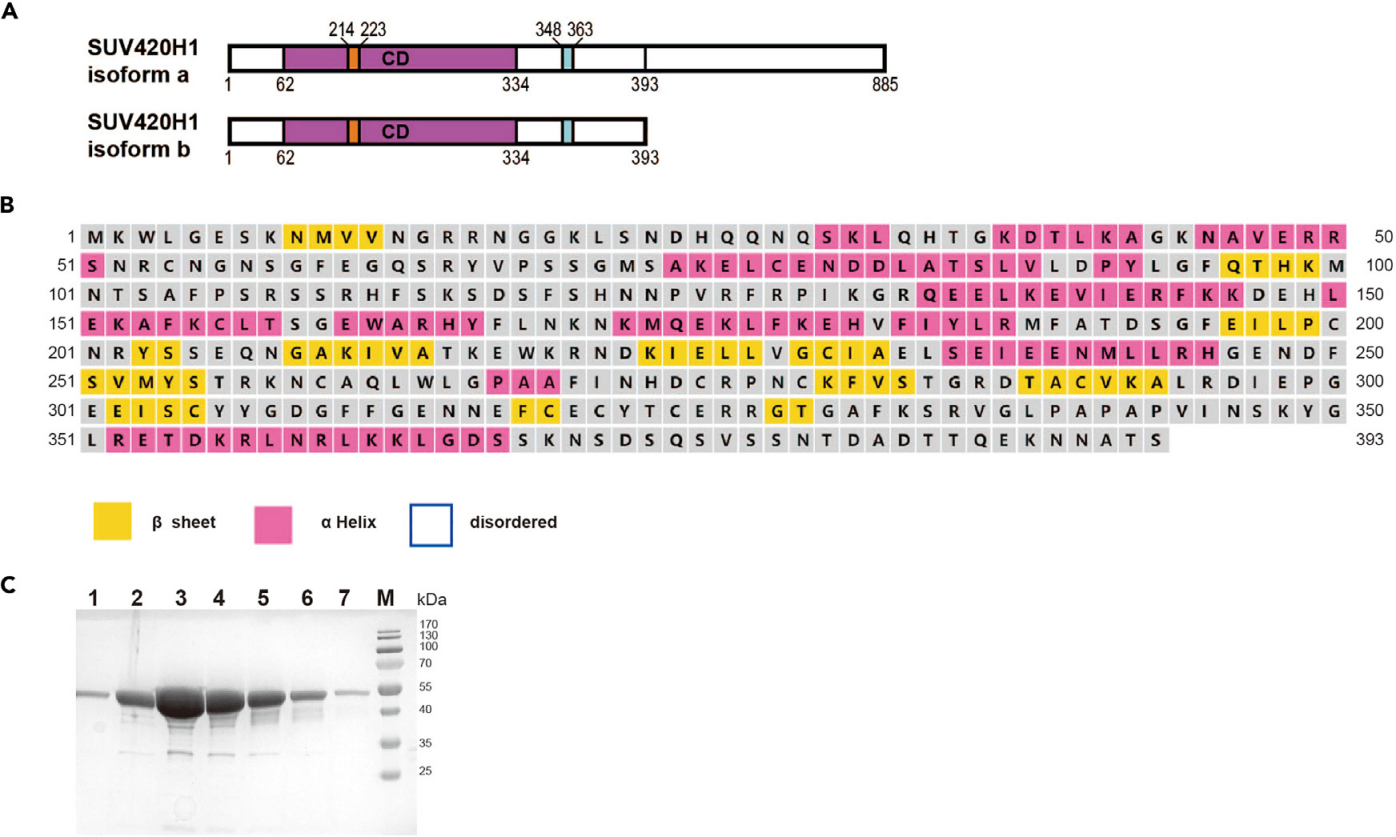
The purification of SUV420H1^1-393^ (A) Schematic illustration of the SUV420H1 isoforms domain architecture. (B) The secondary structure prediction of SUV420H1 (residues 1–393) performed online using PSIPRED 4.0 (http://bioinf.cs.ucl.ac.uk/psipred/). (C) SDS-PAGE analysis of SUV420H1 purified by Superdex 200 gel filtration chromatography.

Human SUV420H1 has two isoforms, SUV420H1 (1–898) and SUV420H1 (1–393),^7^ as shown in Figure 3A. *In vitro* methyltransferase assays demonstrate that SUV420H1 (1–393) not only mediates H4K20me2 deposition into H2A or H2A.Z nucleosomes but also shows a preference for H2A.Z nucleosomes. Secondary structure analysis indicates that the C-terminus of SUV420H1 (1–393) is largely unstructured except for residues 352–367 (Figure 3B). For purification, a 6×His tag was added in-frame at the C-terminus of SUV420H1 and the protein was cloned into the pET17b vector using NdeI and XhoI sites.

1. Transform 1 μL of pET17b-SUV420H1 plasmid into 50 μL of BL21(DE3) RIPL cells as directed by the manufacturer, and plate the cells on LB agar with 100 μg/mL ampicillin and 34 μg/mL chloramphenicol. Incubate at 37°C for 12–16 h.
2. Inoculate a single colony into 100 mL LB medium with ampicillin (100 μg/mL) and chloramphenicol (34 μg/mL), and incubate at 37°C with shaking at 220 rpm for 12–16 h.
3. Dilute 15 mL cell culture into 800 mL LB medium containing ampicillin (100 μg/mL) and chloramphenicol (34 μg/mL), total 4.8 L.
4. Incubate the cell culture at 37°C until it reaches an OD_600_ of 0.6, then cool the culture to 16°C.
5. Add 400 μL of 1 M IPTG to the medium to achieve a final concentration of 0.5 mM and induce SUV420H1 expression for 18 h.
6. Centrifuge the cells at 5000 g for 30 min at 4°C, discard the supernatant, and store the pellet at −80°C for later use.

**Pause point:** The cell pellets can be stored at −80°C for up to 2 months.

7. Resuspend the cell pellet collected from 800 mL in 30 mL of SUV420H1 lysis buffer (20 mM MES pH 6.5, 300 mM NaCl, 5% glycerol, 20 mM imidazole, 5 mM β-mercaptoethanol) and lyse using a high-pressure homogenizer at a cold recycle of 4°C with a pressure of approximately 800 bar for approximately 8 cycles.
8. Centrifuge the lysed cell at 31000 g for 1 h at 4°C. Transfer the clear supernatant with SUV420H1 protein to a new beaker.
9. Equilibrate 3 mL of Ni-NTA resin (QIAGEN) with 60 mL of SUV lysis buffer and mix with the clarified lysate.
10. Incubate the beads and the lysate at 4°C for 1 h, then process through a gravity flow column and wash with 150 mL of SUV lysis buffer.
11. Elute the SUV420H1 protein with about 30 mL SUV elution buffer (20 mM MES pH 6.5, 300 mM NaCl, 5% glycerol, 250 mM imidazole, 5 mM β-mercaptoethanol).
12. Purify the SUV420H1 protein by SP HP cation exchange column.
  a. Dilute the sample into a buffer with final 200 mM NaCl with a half volume of SUV buffer 0 (20 mM MES pH 6.5, 5% glycerol, 5 mM β-mercaptoethanol).
  b. Equilibrate 5 mL SP HP cation exchange column with SUV buffer A (20 mM MES pH 6.5, 200 mM NaCl, 5% glycerol, 5 mM 2-mercaptoethanol) and load the sample onto a 5 mL SP HP cation exchange column.
  c. Elute with a 0.2–1 M NaCl gradient.

***Note:*** The purification process needs to be completed within 1 day to prevent protein degradation.

13. Concentrate the eluted fractions to 500 μL using a 30K MWCO centrifugal filter at 1300 g.
14. Purify the concentrated SUV420H1 proteins using a Superdex 200 gel filtration column in SUV superdex buffer (20 mM MES pH 6.5, 300 mM NaCl, 5% glycerol, 2 mM DTT) and run 15% SDS-PAGE to analyze the peak fractions (Figure 3C). Flash-freeze the purified protein and store at −80°C. Typically, yields of SUV420H1 protein from 6 bottles is 5–10 mg.

**Pause point:** The purified SUV420H1 protein can be stored at −80°C for up to 6 months.

### Installation of the S-ethyl-cysteine (Ecx) into histone H4K20C

**Timing: 2 days**

Alkylation of histone H4K20C with bromoethane results in the formation of the S-ethyl-cysteine (Ecx) mutant protein, H4K20Ecx.

15. We add histone unfolding buffer (20 mM Tris-HCl, pH 8.0, 7 M guanidine hydrochloride, 5 mM β-mercaptoethanol) to achieve a concentration of 2 mg/mL for the H4K20C protein.
16. Mix 500 μL of H4K20C protein solution with 500 μL of alkylation buffer (1.5 M HEPES, pH 8.0, 20 mM D/L-methionine, 20 mM DTT) and reduce the histones for 1 h at 37°C.
17. Add 45 mg of bromoethane (Sigma-Aldrich) as the alkylating agent and incubate at 25°C for 4 h in the dark.

**CRITICAL:** Bromoethane is toxic and it is essential to wear gloves when handling bromoethane. Bromoethane is flammable and keep it away from fire.

**Figure 4:**
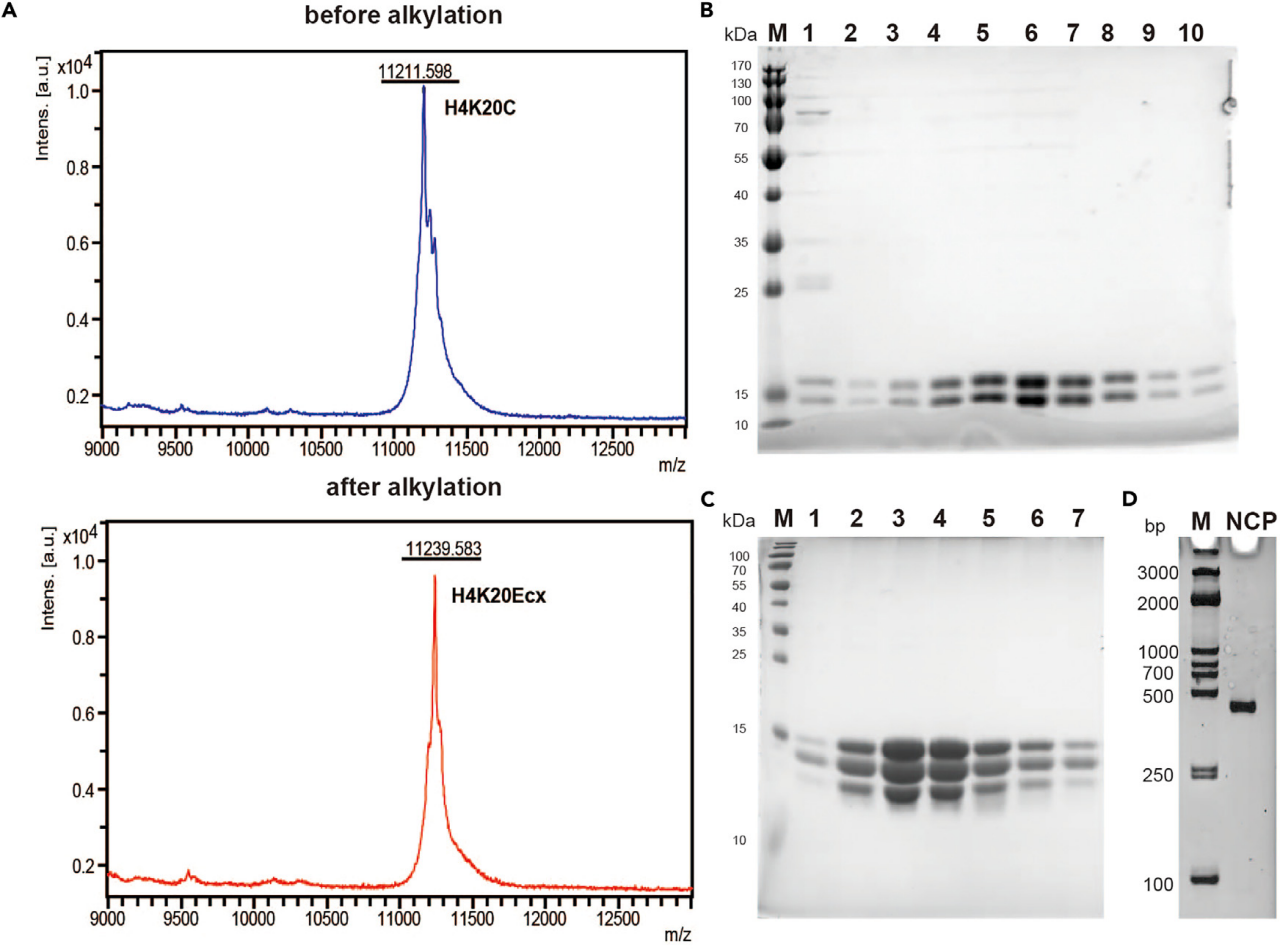
The preparation of H2A.Z-NCP^H4K20Ecx^ (A) Mass spectrum analysis of histone H4 including H4K20Ecx mutation from cysteine alkylation reactions *in vitro*. (B) SDS PAGE analysis of (H3-H4K20Ecx)_2_ purification by SP. (C) SDS PAGE analysis of ZB34 octamer with H4K20Ecx mutant purification by Superdex 200. (D) Native-page analyses of H2A.Z-NCP^H4K20Ecx^ sample.

***Note:*** (1) All of the reactions were performed in the dark. (2) The histones should be 1–10 mg every reaction. (3) Conduct a pre-experiment to confirm the success of the alkylation experiment.

18. Add 10 μL of 1 M DTT to extend the reaction and maintain reducing conditions, continuing at 25°C for over 10 h.
19. Quench the reaction with 50 μL of 14.2 M β-mercaptoethanol and incubate at 25°C for 30 min.
20. Analyze both the modified and unmodified proteins using MALDI-TOF/TOF Ultraflextreme mass spectrometry (Figure 4A).

### Reconstitution of tetramer (H3-H4K20Ecx)_2_

**Timing: 2 days**

The H3 and H4K20ECX proteins are mixed in equimolar ratios and subjected to refolding, resulting in the formation of tetramers.

21. Mix purified H3 and H4K20Ecx proteins in a 1:1 molar ratio.
22. Dialyze the protein mixture against histone refolding buffer (20 mM Tris-HCl pH 8.0, 1 mM EDTA, 2 M NaCl, 5 mM β-mercaptoethanol) thoroughly.
23. Dilute the sample in a buffer with 850 mM NaCl, load onto a 5 mL SP HP cation exchange column equilibrated in histone 34 buffer A (50 mM Tris–HCl, pH 8.0, 1 mM EDTA, 850 mM NaCl, 5 mM β-mercaptoethanol), and elute with a 0.85–2 M NaCl gradient. Run 15% SDS-PAGE to analyze the peak fractions (Figure 4B).
24. Pool the fractions and store at −80°C for further use.

**Pause point:** The purified (H3-H4K20Ecx)_2_ protein can be stored at −80°C for up to 1 year.

### Nucleosome preparation

**Timing: 3 days**

The histone octamer is mixed with DNA in equimolar ratios under high salt conditions, followed by a gradual reduction to low salt conditions, allowing for the stable formation of nucleosomes.

25. Mix purified histones H2A.Z-H2B and (H3-H4K20Ecx)_2_ in a 2:1 M ratio and dialyze against histone refolding buffer (20 mM Tris–HCl, pH 8.0, 2 M NaCl, 1 mM EDTA, 5 mM β-mercaptoethanol) to promote folding into histone octamers for 12–16 h.
26. Load folded histone complexes onto a Superdex 200 gel filtration column equilibrated in histone refolding buffer.
27. Analyze peak fractions using 15% agarose SDS-PAGE (Figure 4C) and collect about 13 mL of peak fractions.
28. Mix purified histone octamers with 147-bp DNA in a 1:1 M ratio in TE2N buffer (20 mM Tris–HCl, pH 8.0, 2 M NaCl, 1 mM EDTA, and 5 mM β-mercaptoethanol), then dialyze with a gradual decrease in NaCl from 2 M to 0.25 M over 18 h, followed by 3 h in TE buffer (20 mM Tris–HCl, pH 7.5, 1 mM EDTA, 5 mM β-mercaptoethanol).

***Note:*** Purified histone octamer must be added at 2 M NaCl condition to prevent disassembly at low salt.

29. Heat-reposition the reconstituted nucleosomes by incubating at 55°C for 2 h.
30. Load the sample onto a DEAE-5PW cation exchange column equilibrated in TE buffer (20 mM Tris–HCl, pH 7.5, 1 mM EDTA, 5 mM β-mercaptoethanol), apply a quick 0–0.4 M KCl gradient (4 mL/min, 10 min) and elute with a 0.40–0.75 M KCl gradient (4 mL/min, 45 min).
31. Collect peak fractions quickly and thoroughly dialyze the purified nucleosomes against HE buffer (20 mM HEPES, pH 7.5, 1 mM EDTA, 5 mM β-mercaptoethanol).
32. Analyze the sample using a 6% Native gel, concentrate to approximately 2 mg/mL, and store at 4°C.

**Pause point:** The purified nucleosomes can be stored at 4°C for up to 3 months.

### Gradient fixation (GraFix)

**Timing: 3 days**

The SUV420H1-nucleosome complex is separated using the GraFix method, while glutaraldehyde is employed for crosslinking, resulting in the formation of stable complexes.

33. Dilute 0.1 mg of the H2A.Z^H4K20ECX^-NCP sample to the concentration of 0.05 mg/mL with complex buffer (HEPES pH 7.5, 4% glycerol, 2 mM DTT). Obtain SUV420H1–H2A.Z^H4K20ECX^-NCP complexes by mixing 0.114 mg of SUV420H1 and H2A.Z ^H4K20ECX^-NCP in a 2.2:1 M ratio. Dialyze against complex buffer for 3 h to remove NaCl.

***Note:*** The SUV420H1 protein must be added at low concentration of nucleosome to prevent largely aggregation.

34. Add 2 μL of 100 mM S-adenosylmethionine (SAM) to into the complex, and incubate for 1 h on ice.

***Note:*** In methylation reactions, SUV420H1 catalyzes the transfer of the methyl group from SAM to H4K20. SAM serves as a methyl donor in methylation reactions. The addition of SAM along with the mutation of H4K20Ecx collectively stabilizes the complex, so the SAM should be added to get sample of high-quality. SAM must be added at low concentration of complex to prevent largely aggregation.

35. Concentrate the sample to achieve a final concentration of 0.5 mg/mL.
36. Create a gradient using 6.2 mL of low sucrose buffer (10 mM HEPES, pH 7.5, 2 mM DTT, 5% sucrose) and 6.2 mL of high sucrose buffer (10 mM HEPES, pH 7.5, 2 mM DTT, 20% sucrose) with 0.15% glutaraldehyde using a gradient master (BioComp) to establish a continuous density and glutaraldehyde gradient (Figure 5).

**Figure 5:**
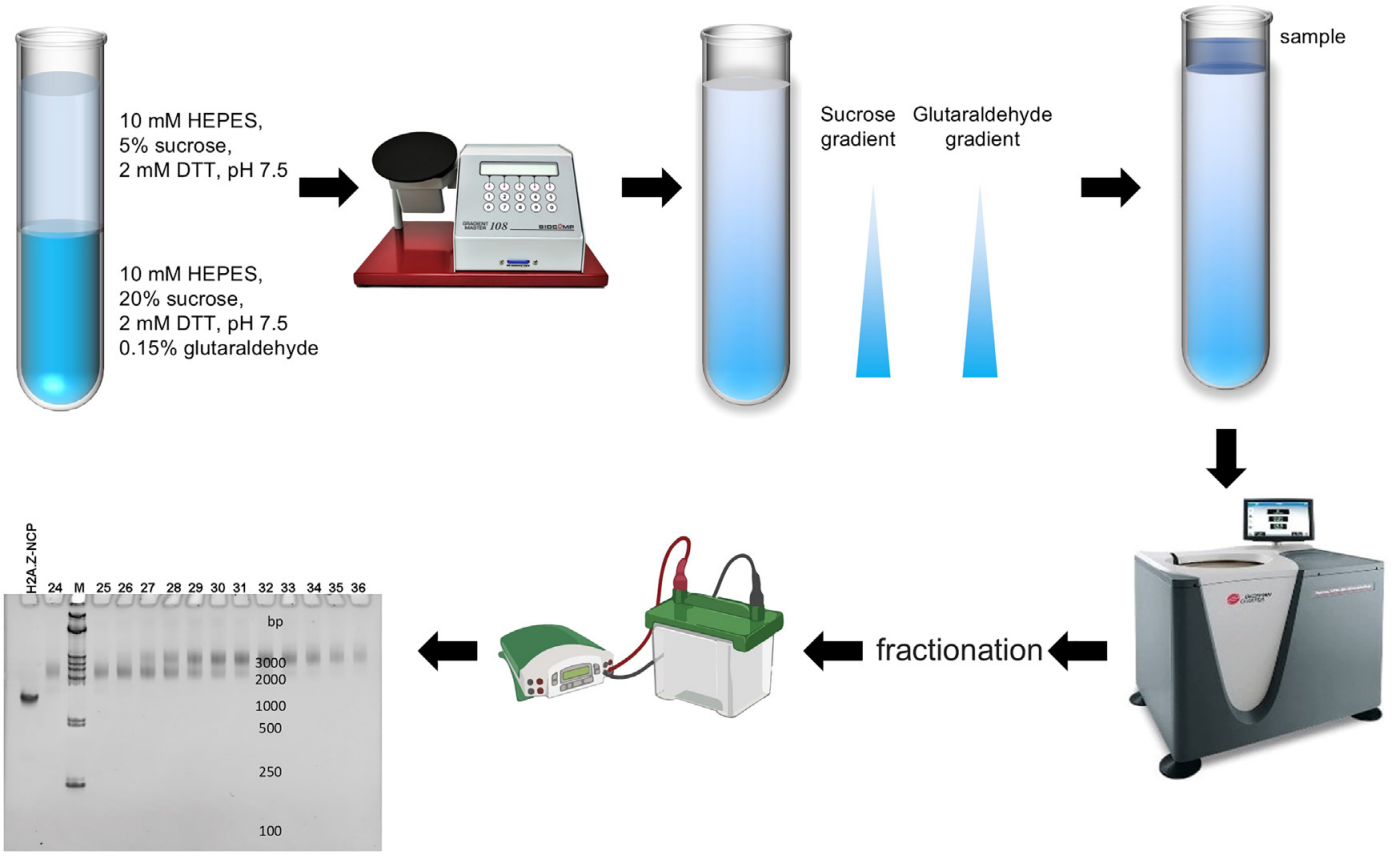
The preparation of SUV420H1-H2A.Z-NCP^H4K20Ecx^ complex
37. Load 200 μL of complexes into the top of the tube. Ultracentrifuge at 4°C for 20 h at 210,100 g using a Beckman SW-41Ti rotor.
38. Collect fractions every 200 μL and analyze them using electrophoresis at 150 V for 60 min on 6.5% native TBE polyacrylamide gels, keeping the gels on ice.
39. Stain the gels with ethidium bromide (EB). Choose the best fractions, dialyze them in HEPES buffer (20 mM HEPES pH 7.5, 2 mM DTT), and concentrate the samples to 0.8 mg/mL for cryo-EM preparation.

**CRITICAL:** Ethidium bromide (EtBr) is highly toxic and can be readily absorbed through the skin. It is essential to wear gloves when handling EtBr solutions.

### Cryo-EM sample check

**Timing: 2 days**

By inspecting the cryo-EM samples, higher-quality samples are selected for data collection. The data is then processed to identify the samples with optimal SUV420H1 binding.

40. Pretreat ANTA copper grids with H_2_/O_2_ gas at 15 mA for 60 s using an easiGlow Glow Discharge Cleaning System.

***Optional:*** The glow-discharge setting may be optimized for different samples.

**Figure 6:**
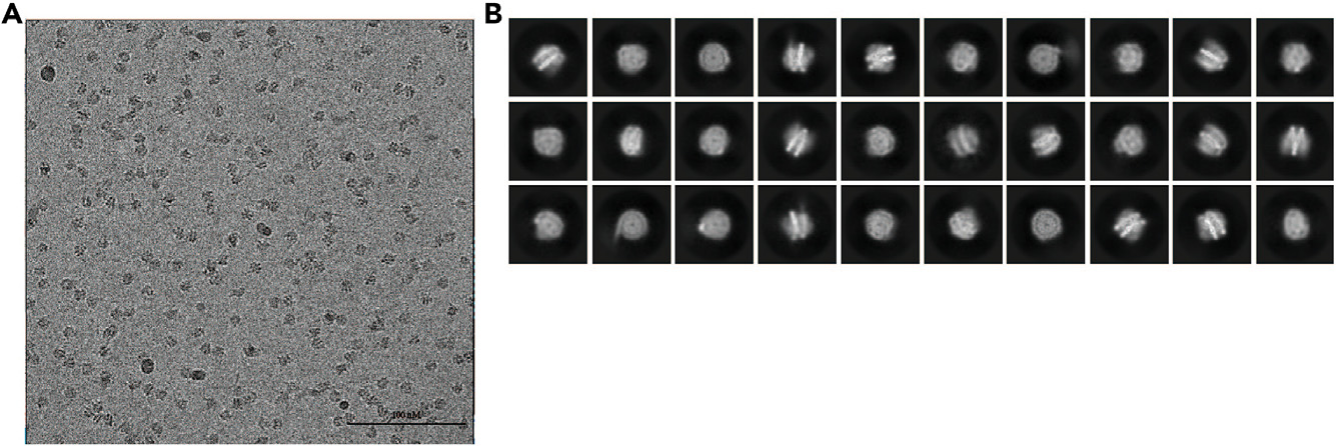
Cryo-EM analysis of SUV420H1-H2A.Z-NCP^H4K20Ecx^ complex (A) The typical cryo-EM micrograph of SUV420H1-H2A.Z-NCP^H4K20Ecx^ complex. (B) Selected 2D class averages from the SUV420H1-H2A.Z-NCP^H4K20Ecx^ complex.

41. Prepare liquid nitrogen-cooled ethane and configure the Vitrobot Mark IV settings to 4°C under 100% humidity.
42. Apply 3.5 μL of the complex sample to the grid, blot for 3 s with blotting force of 2 N and rapidly plunge frozen into cooled liquid ethane using a Vitrobot.
43. Transfer and store the grid in liquid nitrogen.
44. Check the quality of the grid by a Talos Arctica microscope at the Center for Biological Imaging, Institute of Biophysics (Figure 6A) and the binding of SUV420H1 to nucleosome from the 2D class averages (Figure 6B). Optimize the freezing conditions if necessary.

## Expected outcomes

Our method simplifies the incorporation of a lysine analog into recombinant histone H4 by substituting lysine with Ecx. This substitution in H4K20 results in a nucleosome complex with SUV420H1 that is stable enough for near-atomic resolution cryo-EM structural determination. The Ecx modification is an effective chemical strategy for incorporating methyl lysine analogs into low-yield proteins, and applicable for the study of various methyltransferases. A cryo-EM micrograph demonstrating the SUV420H1-H2A.Z^H4K20ECX^ NCP complex is shown in Figure 6. Additionally, this approach has revealed the structure of the methyltransferase SET8 in a complex with a nucleosome.^8^

## Limitations

While the alkylation reaction is straight forward, its application to other amino acids depends on whether substituting cysteine affects protein expression or folding. Proteins with multiple cysteine residues may require additional modifications to prevent unwanted reactions.

## Troubleshooting

### Problem 1

The molar ratio of histone H2A.Z to H2B in the H2A.Z-H2B dimer deviates from 1:1.

### Potential solution

Induce H2A.Z-H2B protein expression by adding IPTG after cooling the culture to 16°C. Confirm that the OD_600_ does not exceed 0.6 at inducing.

### Problem 2

Histone H3 or H4 fail to express.

### Potential solution

Ensure the OD_600_ of the culture does not exceed 0.6 before adding IPTG.

### Problem 3

The sample was either not alkylated or only a minimal portion was alkylated.

### Potential solution

Make sure the pH of alkylation buffer is 7.5 and the DTT solution is effective.

### Problem 4

The mixture of SUV420H1 and H2A.Z-NCP forms cloudy precipitate.

### Potential solution

Dialyze against a salt-free buffer rather than centrifuging. This may help to eliminate aggregation due to complex instability in salty buffer.

### Problem 5

The cryo-EM screen reveals a lot of nucleosomes without SUV420H1.

### Potential solution

The glutaraldehyde crosslink may be unstable upon removal. Freeze the sample immediately after changing the buffer to preserve the complex

## Resource availability

### Lead contact

Further information and requests for resources and reagents should be directed to and will be fulfilled by the lead contact, Zheng Zhou (zhouzh@ibp.ac.cn).

### Technical contact

Technical questions on executing this protocol should be directed to and will be answered by the technical contact, Li Huang (huangli@moon.ibp.ac.cn;).

### Materials availability

Plasmids generated in this study will be made available on request, but we may require a payment and/or a completed Materials Transfer Agreement if there is potential for commercial application.

### Data and code availability

This protocol did not generate datasets or code.

## Acknowledgments

We thank Zhao Weiwei from the China State Institute of Pharmaceutical Industry for their assistance in designing the Ecx mutation experiments. We thank Dr. Xiaoli Feng for assistance in experiments and members in Core Facilities for Protein Science at the Institute of Biophysics, Chinese Academy of Science (IBP, CAS) for technical help. This work was supported by grants from the 10.13039/100014717Natural Science Foundation of China (32320103008, 32270651, and 32370642), the Ministry of Science and Technology of China (2021YFA1300100), and the 10.13039/501100002360Chinese Academy of Sciences (CAS) Strategic Priority Research Program (XDB37010100).

## Author contributions

L.H. designed and performed the experiment. L.H. wrote the manuscript. Z.Z. edited the manuscript.

## Declaration of interests

The authors declare no competing interests.
